# Osteocytes contribute to sex-specific differences in osteoarthritic pain

**DOI:** 10.3389/fendo.2024.1480274

**Published:** 2024-11-07

**Authors:** Ryan Jones, Sophie J. Gilbert, Sarah R. Christofides, Deborah J. Mason

**Affiliations:** Biomechanics and Bioengineering Research Centre Versus Arthritis, School of Biosciences, Cardiff University, Cardiff, United Kingdom

**Keywords:** osteoarthritis, pain, osteocyte, sex differences, menopause

## Abstract

Osteoarthritic (OA) pain affects 18% of females and 9.6% of males aged over 60 worldwide, with 62% of all OA patients being women. The molecular drivers of sex-based differences in OA are unknown. Bone is intricately coupled with the sensory nervous system and one of the only joint tissues known to show changes that correlate with patient pain in OA. There are fundamental sex differences in pain sensation and bone biology which may be intrinsic to OA disease progression, however these differences are vastly under researched. We have utilised three data sets to investigate the hypothesis that potential mediators responsible for sex dependent pain mechanisms displayed in OA are derived from mechanically stimulated osteocytes. Our published dataset of the *in vitro* human osteocyte mechanosome was independently compared with published data from, sex-based gene expression differences in human long bone, the sex-based gene expression differences during the skeletal maturation of the mouse osteocyte transcriptome and sex specific OA risk factors and effector genes in a large human GWAS. 80 of the 377 sex-specific genes identified in the mouse osteocyte transcriptome were mechanically regulated in osteocytes with enrichment associated with neural crest migration and axon extension, and DISEASES analysis enrichment for the rheumatoid arthritis pathway. 3861 mechanically regulated osteocytic genes displayed sex-specific differences in human long bone with enrichment for genes associated with the synapse, sensory perception of pain, axon guidance, immune responses, distal peripheral sensory neuropathy, sensory neuropathy, and poor wound healing. 32 of 77 effector genes and 1 of 3 female specific OA risk factor genes identified in the human GWAS were differentially expressed in the osteocyte mechanosome and male and female bone. This analysis lends support to the hypothesis that mechanically regulated genes in osteocytes could influence sex specific differences in osteoarthritic pain and highlights pain pathways with approved drugs that could potentially treat elevated pain susceptibility in females with OA.

## 1 Introduction

Chronic pain in osteoarthritis (OA) is a severe and debilitating condition affecting an estimated 530 million sufferers worldwide, limiting patient mobility, ability to perform daily activities and live independently (1). Sex-based differences in the clinical presentation and prevalence of OA has been described for decades but are widely under-researched (2). Women over the age of 55 have a higher prevalence of knee OA than men of the same age (3) and a higher prevalence of hand OA (4). Women with OA also suffer more debilitating pain (3), more annual articular cartilage loss (5) and have a more severe radiographic OA when compared to equivalent male patients. Female sex hormones, such as oestrogen, are known to act directly on nociceptors to mitigate pain (6) and exert protective roles in articular cartilage and subchondral bone (5, 7) and more than half of post-menopausal women suffer with OA pain (8). Therefore, it has been assumed that the differences in male and female OA pain and progression are due to withdrawal of the protective effects of oestrogen (9) but research results are controversial. In a large cohort study, Cirillo et al. found that oestrogen treatment alone lowered the prevalence of hip replacement but not knee replacement (10) whereas another study of post-menopausal woman with both symptomatic and radiographic OA receiving hormone therapy, reported a lower prevalence of knee OA (11).

Bone is sexually dimorphic, displaying different gene expression profiles, sex hormone sensitivities and mechanical responses between males and females. Subchondral bone is also one of the only tissues to show structural changes that correlate to pain in OA patients (12). The inhibition of bone resorption to prevent OA disease progression in animal models and OA patients is also an emerging research area (reviewed in 13). There are intimate associations between nerves and bone. Skeletal sensory nerve sprouting, invasion and sensitisation are associated with bone pathologies, demonstrated in rodent and human OA joints, and intrinsic to pain responses in animal models of OA (14), and humans with OA (15). Densely innervated subchondral bone channels have been shown to accompany sclerotic subchondral bone remodelling in the tibial plateaux and femoral condyles of end-stage OA knees in animal models (16). Areas of OA structural damage, necrosis and remodelling in the subchondral bone known as bone marrow lesions (BML) are characterised by sensory nerve invasion and correlate with pain (17). Enlargement of BMLs is associated with worsening joint degeneration and increased pain, whereas reduced BML severity relieves pain (18). The formation of BMLs is known to be modulated by mechanical loading. Abnormal joint loading through obesity, malalignment, trauma, or joint instability are key risk factors for OA (19). BML presence and location are associated with joint malalignment (20); medial BMLs occur mostly in varus knee, lateral in valgus (20). Absence/regression of BMLs occurs following mechanical (bracing) (21) or bone sparing pharmaceutical (Zoledronic acid) interventions (22). Osteocytes, the mechanosensing cells in bone, orchestrate bone remodelling in response to mechanical load, inflammation, and hormones (23). In a previous study, we developed a human 3D model of osteocytes differentiated from Y201 Mesenchymal Stem Cells in Type I collagen (24). This model shows dendritic morphology, and expresses osteocyte markers BGLAP SOST, PDPN, OPG, GJA1, C44, FGF23, PHEX and PHOSPHO1 (24). Pathophysiological (4300 microstrain) (25) loads applied to this osteocyte model under osteogenic conditions regulated proteins reflecting bone remodelling and inflammation. RNAseq analysis on pathophysiologically loaded versus unloaded osteocytes in this 3D model revealed 7564 differentially expressed genes (DEGs), which we have called the osteocyte mechanosome (24). The osteocyte mechanosome included genes involved in inflammation, matrix organisation, ageing, ossification, bone morphogenesis, cartilage development, and bone mineralisation (24) as well as > 200 genes directly involved in nociception, neuropathic pain, nociceptor sensitisation, neuronal axonal guidance, and neuro-sensitivity (24). We have previously used this model to investigate mechanical and inflammatory mechanisms underlying osteoarthritic pathology (24). In the current hypothesis and theory paper, we have compared published data with the osteocyte mechanosome to test the notion that genes that are mechanically regulated in osteocytes and differentially expressed in males and females could explain sex-specific susceptibility to pain.

We hypothesise that differences between male and female susceptibility to osteoarthritic pain is influenced by sex-specific responses of osteocytes to mechanical stimulation. To test this, we have performed a meta-analysis of published RNAseq data to determine whether regulated genes in the osteocyte mechanosome are differentially expressed in male and female bone. The resulting sex-specific mechanically regulated genes were compared with OA risk loci from human Genome Wide Association Studies (GWAS) to highlight mediators linked to sex differences in OA. We then investigate whether these sex specific genes in the osteocyte mechanosome are associated with pathways linked to the generation of pain and represent new druggable targets that could treat female heightened susceptibility to osteoarthritic pain.

## 2 Methods and results

**Figure 1:**
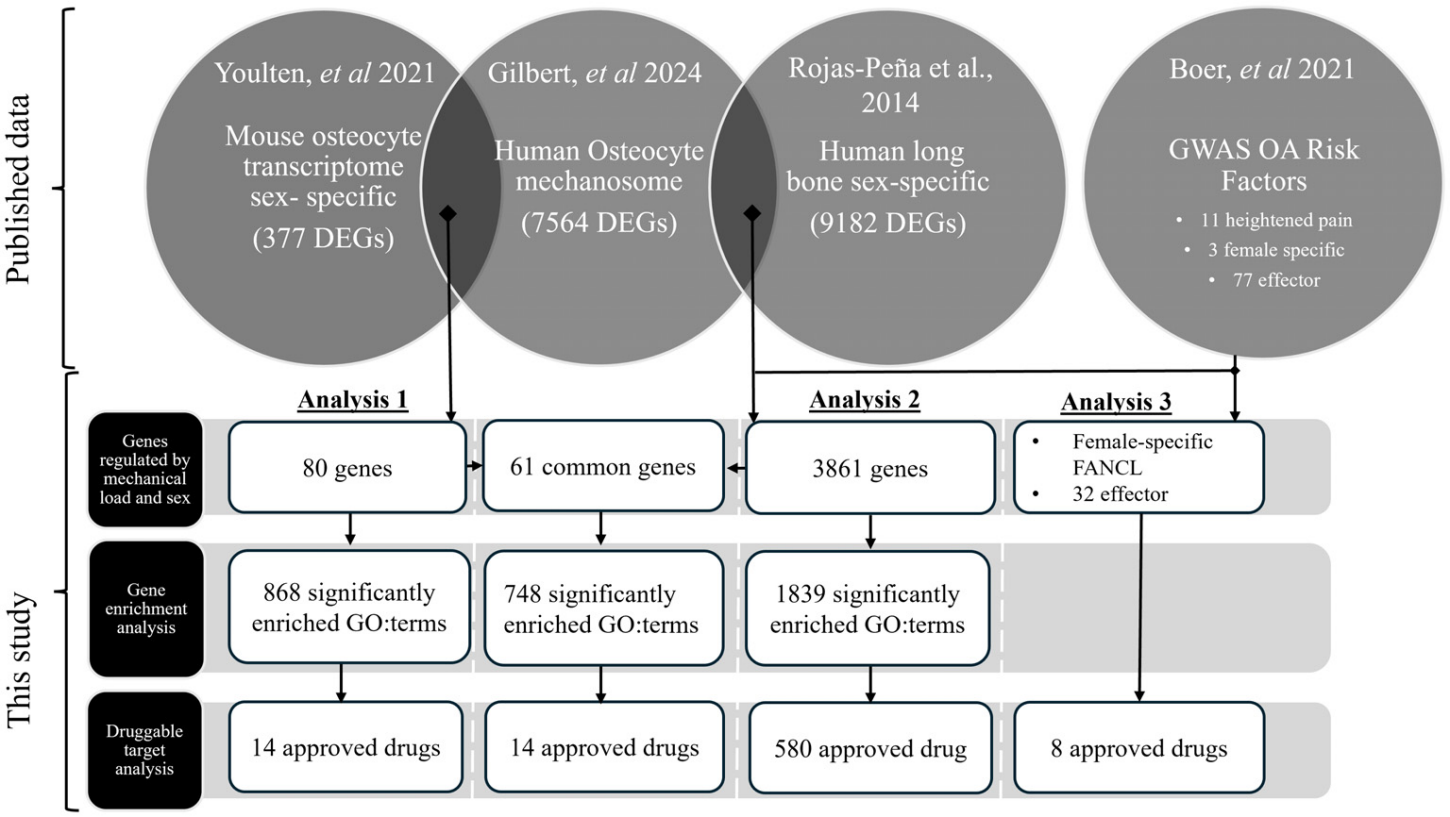
RNA sequencing data from mechanically regulated osteocytes (‘the osteocyte mechanosome’) (24) were independently compared with sex specific differences identified in the mouse osteocyte transcriptome (26) (Analysis 1) and sex-specific human long bone derived osteoblasts (27) (Analysis 2), and genes that showed co-regulation taken forward for further analysis. Mechanically regulated genes in the osteocyte mechanosome that also displayed sex regulation in the human long bone derived osteoblasts (Analysis 2) were combined with human GWAS loci associated with increased risk of OA in females and heightened OA pain (28) (Analysis 3). Sex specific mechanically regulated genes in osteocytes were searched for functional and disease pathway enrichment (29) and druggable targets (30).

Three independent analyses were used to interrogate the above hypothesis; these were then combined to investigate the potential mediators responsible for the osteocyte-derived sex dependent pain mechanisms displayed in OA (**Figure 1**). All analysis was performed using R 4.3.1 (31) in RStudio 2023.12.0 (32). Our published dataset (24) of *in vitro* human osteocyte responses to pathophysiological mechanical loading (‘osteocyte mechanosome’) was independently compared with published data from, sex-based gene expression differences during the skeletal maturation of the mouse osteocyte transcriptome (Analysis 1) (26), the sex-based gene expression differences in human long bone explant-derived osteoblasts (Analysis 2) from 4 healthy children (a reanalysis of a subset of published dataset in Sex-Associated Gene Database repository number 00129 (27, 33) and sex specific OA risk factors and effector genes in a large human GWAS of 826,690 individuals from 9 populations (Analysis 3) (28). The osteocyte transcriptome and SAGD datasets were selected as they represent the only available RNA sequencing datasets detailing sex-regulated gene expression within bone. The GWAS data was selected as the largest OA GWAS currently available worldwide. Log2 fold changes (log2FC) were standardised so that females were always the numerator and males the denominator (*i.e.*, a positive log2FC would correspond to higher expression in females, and a negative log2FC to a higher expression in males. Positive log2FC within the osteocyte mechanosome indicates genes upregulated by mechanical load, whereas negative log2FC indicates downregulation in response to loading.

Protein encoding genes that displayed significant regulation by mechanical loading in the osteocyte mechanosome and by sex in published datasets (Analyses 1 and 2) were used to identify potential mediators of sex-based differences in OA pain (**Figure 1**). GWAS loci associated with heightened OA pain, female specific risk factors and OA effector genes (28) was combined with the sex-specific mechanically regulated genes identified from Analysis 2 to reveal sex specific OA risk factors in the osteocyte mechanosome (Analysis 3) (**Figure 1**).

The resulting genes from each analysis that displayed significant co-regulation were annotated with GO terms from Ensembl. Over-representation analysis was performed using clusterProfiler (29) and GO.db (34), using all genes represented in the osteocyte mechanosome as the enrichment background. Computational prediction of protein-protein network interactions of significantly regulated genes was performed using String.db (35). The top 1000 protein-protein interactions were generated in R (31) then uploaded to the string online interface (string-db.org) for STRING network analysis. Gene Ontology, KEGG pathway, human phenotype (Monarch) Disease-gene association (DISEASES) and annotated keyword (UniProt) functional enrichment analyses of the generated network were also performed using this online interface. GO:terms known to be affiliated with the generation of pain responses or joint pathology that were enriched within the dataset were extracted and used to highlight mediator genes in biplot graphs and tables. Within individual biplots, all genes beyond a set Log2-fold change threshold (**Supplementary Table 1**) were labelled to ensure graph clarity and specific genes of interest outside this range italicised. Sex specific genes in the osteocyte mechanosome were searched for druggable targets (30) (https://www.dgidb.org).

### 2.1 Analysis 2 - the osteocyte mechanosome and sex specific differences in human long bone derived osteoblasts

In Analysis 1 (**Figure 1**), our osteocyte mechanosome was combined with a published dataset from within the osteocyte transcriptome reflecting DEGs in mouse male and female osteocytes (26). Differentially expressed osteocyte enriched genes were identified from bones of skeletally mature (16 weeks) and aged (26 weeks) male and female mice. The data from Youlten et al. (26) was downloaded from the associated GitHub repository (26) and their analysis recapitulated using their code to regenerate a list of genes associated with the osteocyte transcriptome. In brief, differential expression between male and female mice in their dataset was calculated just for osteocyte-associated genes using *edgeR* (36) and *limma* (37). Sex comparisons were carried out separately for 16-week-old and 26-week-old mice.

**Figure 2:**
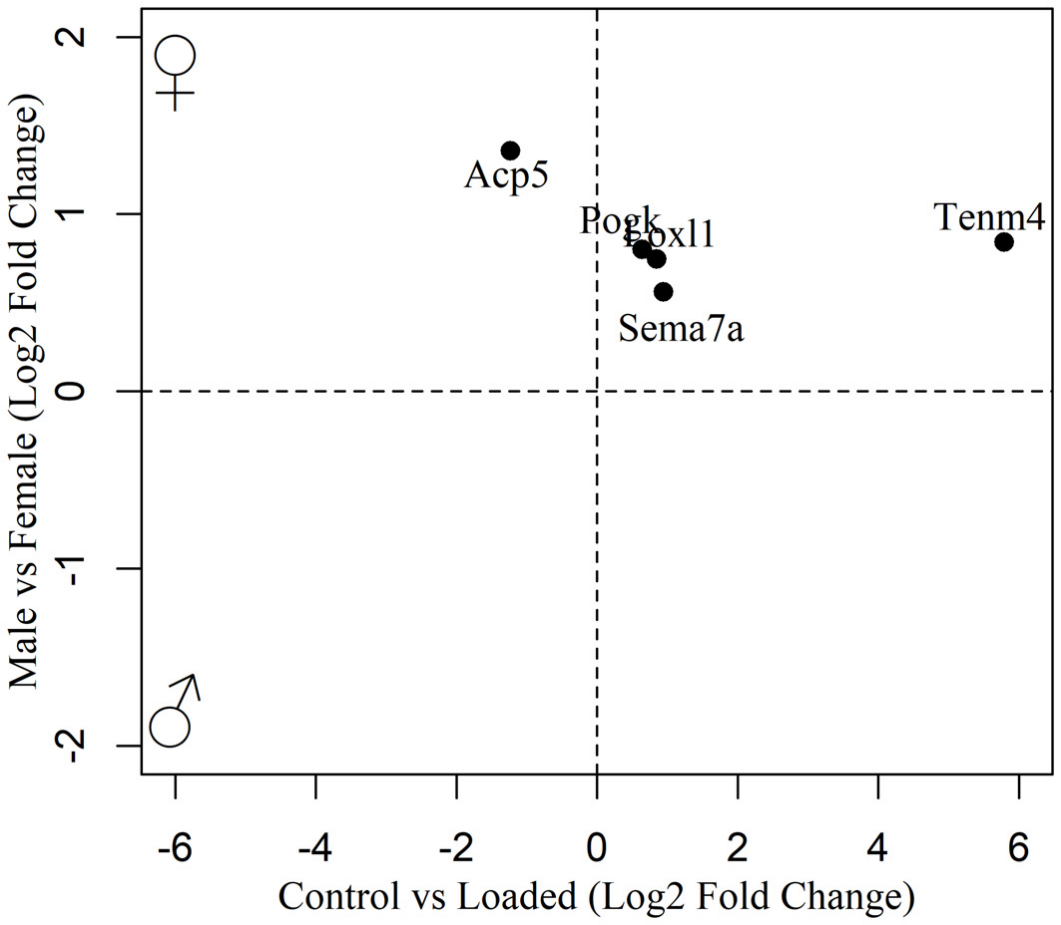
Biplot of genes regulated in the osteocyte mechanosome and differentially expressed in osteocytes from male and female 16-week-old skeletally mature mice.

**Figure 3:**
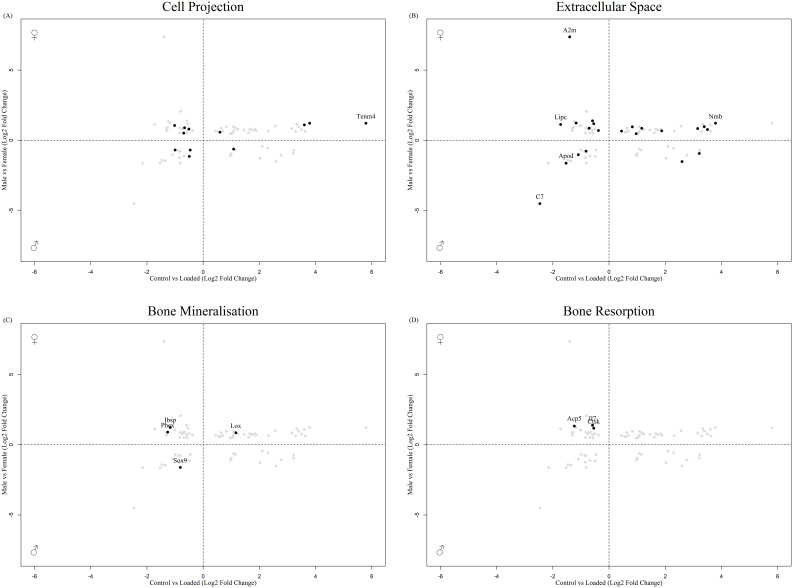
Biplot of genes regulated in the osteocyte mechanosome and differentially expressed in osteocytes from male and female 26-week-old mice. Points highlighted by GO Enrichment analysis. **(A)** Cell projection, **(B)** Extracellular space, **(C)** Bone mineralisation, **(D)** Bone resorption.

In total, 80 DEGs in the osteocyte mechanosome were also differentially expressed in mouse osteocytes from males and females at either 16 (**Figure 2**, **Supplementary Table 2**) or 26 (**Figure 3**, **Supplementary Table 3**) weeks of age. In 16-week-old mice, 5 osteocyte mechanosome DEGs also displayed differential expression between males and females: TENM4, SEMA7A, LOXL1, POGK and ACP5 (**Figure 2**). GO term enrichment of these genes was associated with collagen-containing extracellular matrix and bone morphogenesis and resorption, neural crest migration, and positive regulation of axon extension (**Supplementary Figure 1**). In contrast, in aged, 26-week-old mice, 77 DEGs in the osteocyte mechanosome showed significant differences between male and female osteocytes (**Figure 3**). Of note, these included TENM4, LOX and CTSK all of which displayed higher expression in females and mechanical regulation (**Figure 3**, **Table 1**).

**Table 1:** Pathways significantly enriched in the top 1000 protein-protein interactions in the genes regulated in the osteocyte mechanosome and by sex in human long bone derived osteoblasts.

Group	Analysis	Pathway	Count	Strength	fdr
Top 1000 protein-protein interactions in genes significantly regulated in Analysis 1	**KEGG**	rheumatoid arthritis	13 of 83	0.49	0.0129
		TGF-β signalling	14 of 91	0.48	0.0108
		complement and coagulation cascades	14 of 82	0.53	0.0056
	**DISEASES**	bone disease	54 of 540	0.29	0.0066
		neurodegenerative disease	47 of 481	0.28	0.024
		musculoskeletal disease	106 of 1154	0.26	<0.0001
		nervous system disease	161 of 2275	0.14	0.0145
	**Monarch**	severe generalised osteoporosis	6 of 11	1.03	0.0034
		distal peripheral sensory neuropathy	5 of 12	0.91	0.0207
		sensory neuropathy	14 of 85	0.51	0.0078
		osteolysis of the upper limb	7 of 19	0.86	0.0053
		osteolysis	12 of 73	0.51	0.0078
		poor wound healing	7 of 20	0.84	0.0064
	**Annotated Keywords (Uniprot)**	osteogenesis imperfecta	8 of 83	0.26	0.0022
		Charcot-Marie-Tooth	10 of 55	0.55	0.0159
		neuropathy	17 of 108	0.49	0.0027
		angiogenesis	20 of 131	0.48	0.0013
Largest cluster of protein interactions	**DISEASES**	degenerative disc disease	3 of 13	1.83	0.0081
	**TISSUES**	rheumatoid arthritis synovial tissues	3 of 9	1.98	0.00056

GO:term enrichment revealed that DEGs in the osteocyte mechanosome and regulated by sex in aged mice bones were predominantly associated with cell projection, extracellular space, bone mineralisation and bone resorption (**Supplementary Figure 2**). No genes associated with the nerve growth factor signalling pathway GO terms were significantly regulated in the dataset.

STRING analysis of all protein interactions that were significantly sex regulated in osteocyte signature and in the osteocyte mechanosome produced a protein interaction network with 25 predicted functional associations compared to the number of expected interactions of 11 (**Supplementary Figure 3**). Functional enrichment analysis of the network showed a protein-protein interaction enrichment P value of < 0.001 and pathways relevant to pain in OA. KEGG pathway analysis revealed the rheumatoid arthritis pathway (count 4 of 83, strength 1.23, fdr 0.0195) and osteoclast differentiation pathway (count 4 of 120, strength 1.07, fdr 0.0280) were enriched in this protein interaction network.

### 2.2 Analysis 2 - the osteocyte mechanosome and sex specific differences in human long bones

In Analysis 2 (**Figure 1**), our osteocyte mechanosome was compared with a published dataset containing 9182 DEGs between human male and female long bone explant-derived osteoblasts downloaded from the sex-associated gene database (repository number 00129; SAGD http://bioinfo.life.hust.edu.cn/SAGD, (27, 33).

**Figure 4:**
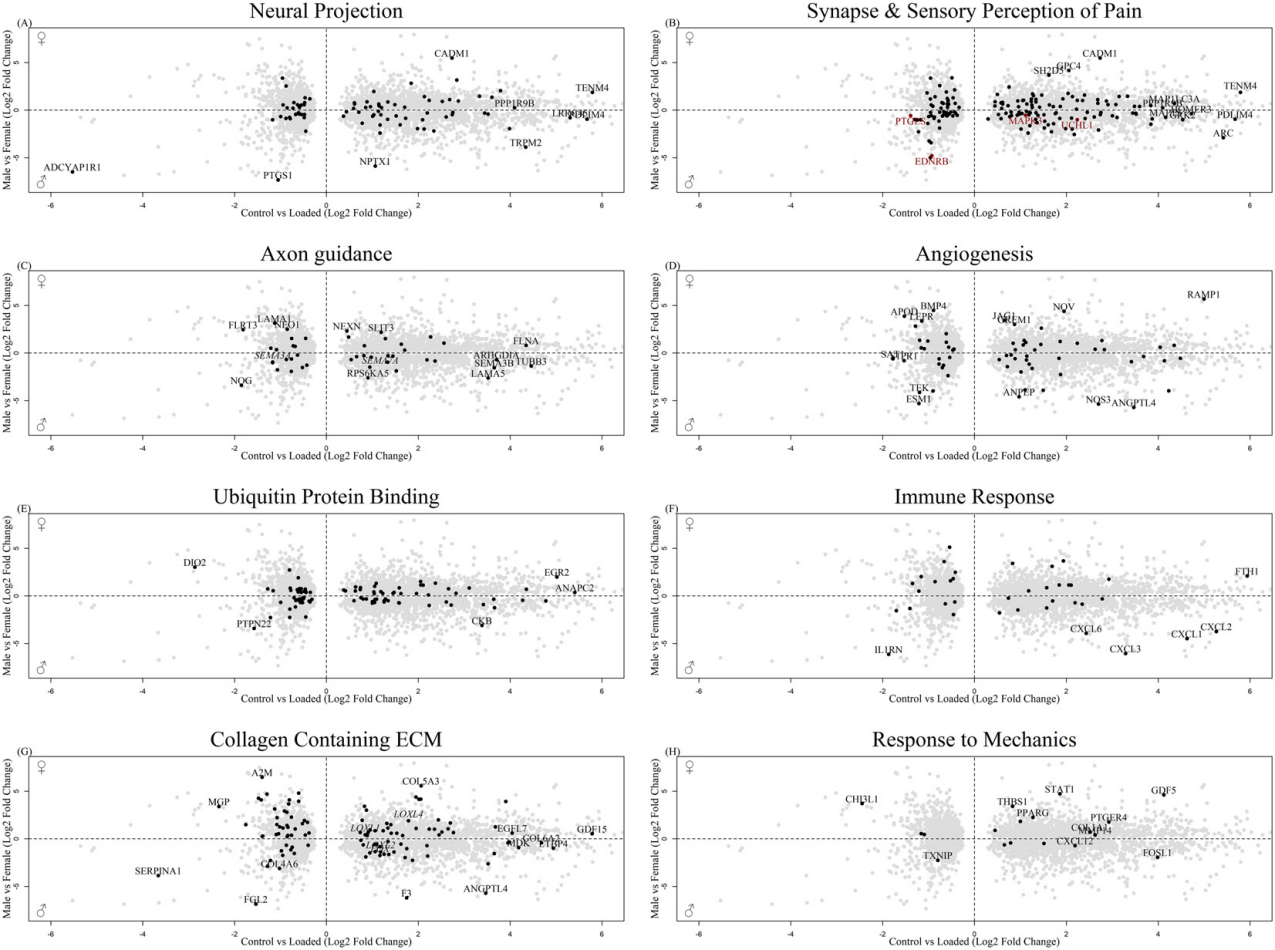
Biplot of genes regulated in the osteocyte mechanosome and differentially expressed in male and female human long bone derived osteoblasts. Points highlighted by GO: Term. **(A)** Neural Projection, **(B)** Black = Synapse, Red = Sensory perception of pain **(C)**. Axon guidance, **(D)** Angiogenesis, **(E)** Ubiquitin protein binding **(F)** Immune response, **(G)** Collagen-containing extracellular matrix **(H)** Response to mechanical stimulus.

3861 of those sex-regulated genes were affected by mechanical load in the osteocyte mechanosome (**Supplementary Table 4**). GO:term enrichment showed that these co-regulated genes are associated with numerous biological processes including those relevant to mechanical loading of bone and pain, such as collagen containing extracellular matrix (ECM), signal transduction, synapse, neural projection, angiogenesis and integrin, cadherin, and calcium ion binding (**Supplementary Figure 3**, **Supplementary Table 5**). The co-regulated genes included 86 associated with neural projection including TENM4 (**Figure 4A**), 155 associated with the synapse (**Figure 4B**) 4 genes associated with the sensory perception of pain including PTGES, EDNRB and 8 members of the MAPK signalling pathway (**Figure 4B**, red), and 44 genes associated with axon guidance including, SEMA3A and SEMA7A (**Figure 4C**). In addition, co-regulated genes included 88 genes associated with angiogenesis including NOS3 (**Figure 4D**), 93 genes associated with ubiquitin protein ligase binding (**Figure 4E**), 39 genes associated with immune responses (**Figure 4F**) and 108 genes associated with collagen containing ECM (**Figure 4G**). Out of the 11 GO terms for mechanical regulation, the GO term ‘response to mechanical stimulus’ identified 17 DEGs in the osteocyte mechanosome that were also differentially regulated in male and female long bones (**Figure 4H**).

**Figure 5:**
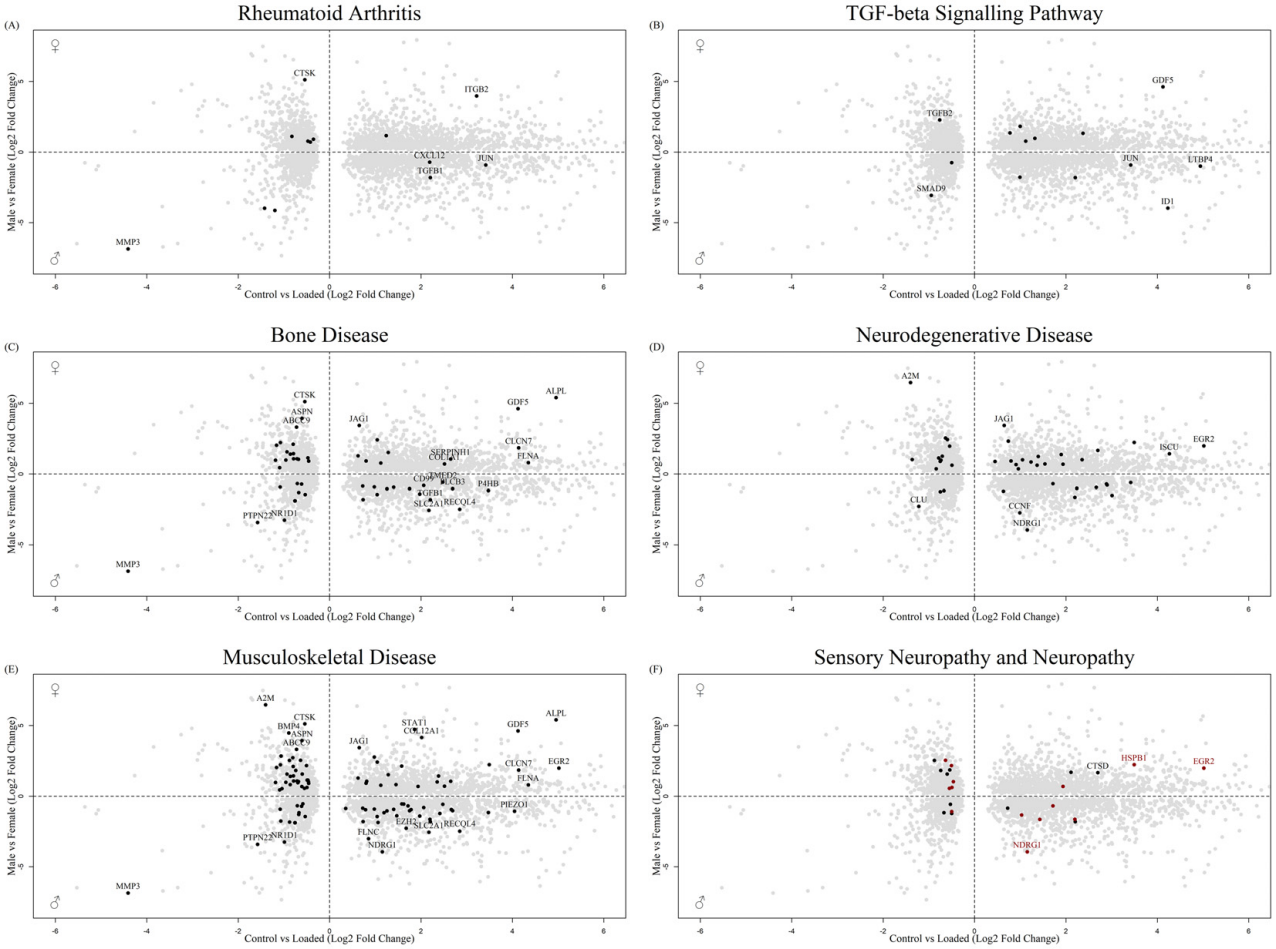
Biplot of genes regulated in the osteocyte mechanosome and differentially expressed in male and female human long bone derived osteoblasts. Points highlighted by STRING Enrichment analysis. **(A)** Rheumatoid arthritis, **(B)** TGF-beta signalling pathway, **(C)** Bone disease, **(D)** Neurodegenerative disease, **(E)** Musculoskeletal disease, **(F)** Black = Sensory neuropathy & Red = neuropathy.

STRING analysis was performed on the top 1000 protein interactions that were significantly sex regulated in human long bone derived osteoblasts and in the osteocyte mechanosome. This produced a protein interaction network with 8786 predicted functional associations (**Supplementary Figure 2**). Functional enrichment analysis of the network showed significant protein-protein interaction enrichment (P < 0.001) and numerous pathways relevant to pain in OA (**Table 1**). Biological process Gene Ontology enrichment showed a very large number of overrepresented pathways: the highest enrichment was for angiogenesis and blood vessel related genes, including retinal blood vessel morphogenesis, data not shown. The most enriched KEGG pathways were associated with the cell cycle, metabolism, and ECM interactions. Interestingly, the rheumatoid arthritis pathway (**Figure 5A**), transforming growth factor-β (TGF-β) signalling (**Figure 5B**) and complement and coagulation cascades all showed enrichment. Disease-gene associations (DISEASES) pathway analysis of co-regulated genes identified bone disease (**Figure 5C**), neurodegenerative disease (**Figure 5D**), musculoskeletal disease (**Figure 5E**), and nervous system disease. Human phenotype (Monarch) analysis of the protein interactions within this dataset revealed enrichment of severe generalised osteoporosis, distal peripheral sensory neuropathy (**Figure 5F**, Black), sensory neuropathy (**Figure 5F**, Red), osteolysis of the upper limb, osteolysis, and poor wound healing. Analysis of the Annotated Keywords (Uniprot) of the protein interaction network revealed enrichment in pathways of osteogenesis imperfecta, Charcot-Marie-Tooth, Neuropathy, and angiogenesis. MCL clustering of the protein interaction network produced 292 clusters, 29 of which included more than 5 genes. The largest cluster was compiled of 68 genes including CXCL12, CTSK and MMP1-3. and was predominantly associated with degenerative disc disease in disease -gene associations (DISEASES) enrichment and included rheumatoid arthritis disease specific synovial tissues in Tissue expression (TISSUES) analysis (**Table 1**).

**Figure 6:**
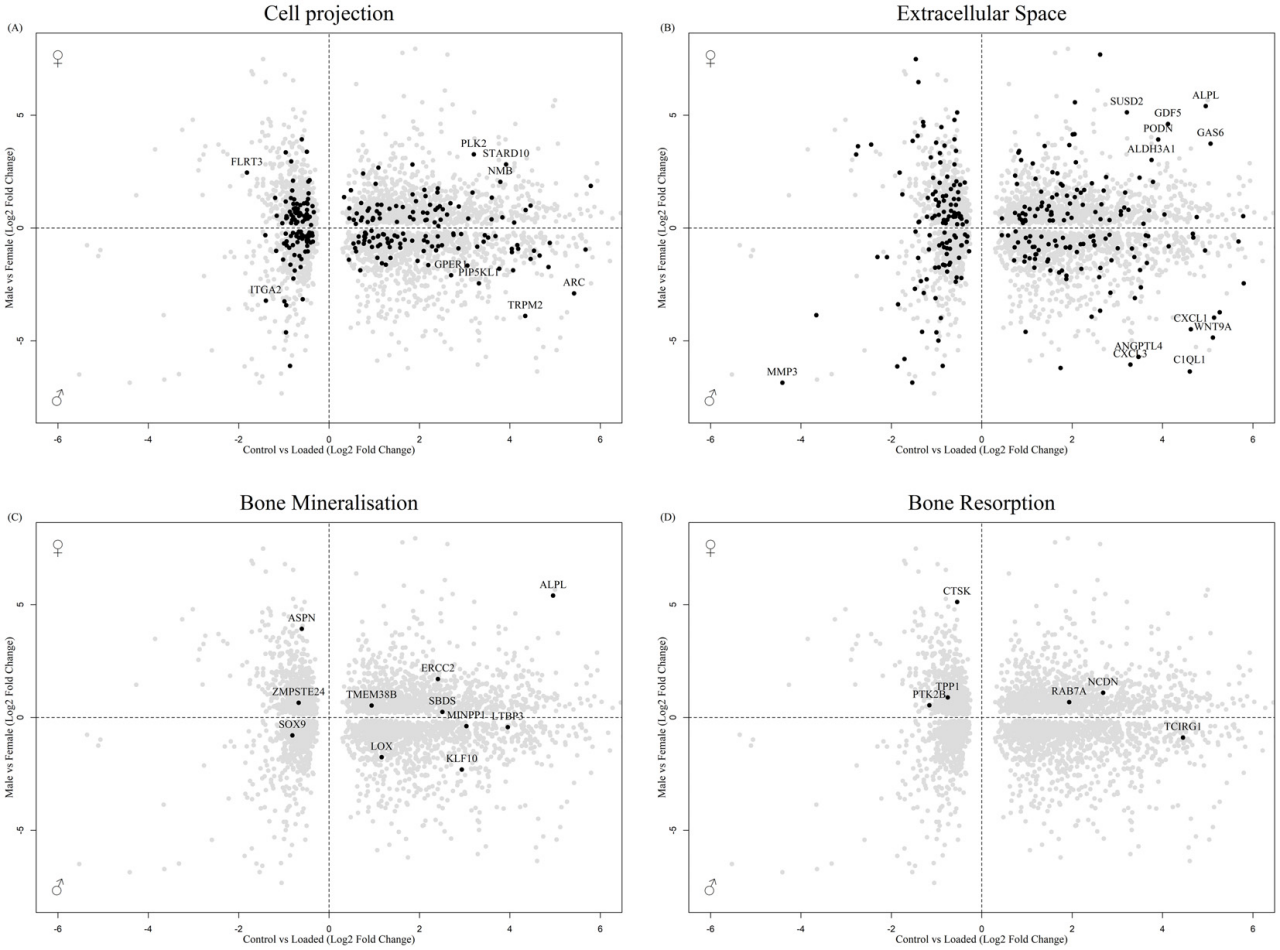
Biplot of genes regulated in the osteocyte mechanosome and differentially expressed in male and female human long bone derived osteoblasts. Points highlighted by GO Enrichment analysis. **(A)** Cell projection, **(B)** Extracellular space, **(C)** Bone mineralisation, **(D)** Bone resorption.

Analysis 2 revealed numerous genes associated with cell projection (**Figure 6A**), extracellular space (**Figure 6B**) and bone biology (**Figures 6C, D**), including ASPN and CTSK, that were regulated by mechanical load in the osteocyte mechanosome and by sex. Members of the LOX pathway showed upregulation by mechanical loading in the osteocyte mechanosome and regulation by sex in human long bones. LOX and LOXL2 were increased in males. LOXL1 and LOXL4 conversely were increased in females. No genes associated with the nerve growth factor signalling pathway GO:terms were significantly regulated in the combined dataset. The fold changes and p values of individual genes of interest selected from these GO:term analyses can be found in **Table 2**.

**Table 2:** Genes regulated in the osteocyte mechanosome and by sex in human long bone derived osteoblasts identified as potential mediators linked to pain generation in OA pathology.

Gene	Mechanosome		Human long bone	
	log2FC	P(adj)	log2FC	P(adj)
TENM4	5.79	0.001	1.22	0.0144
PTGES	-1.39	0.006	-0.99	<0.0001
EDNRB	-0.929	0.045	-4.73	<0.0001
UCHL1	2.24	0.0308	-1	<0.0001
SEMA3A	-1.17	<0.0001	-0.949	<0.0001
SEMA7A	0.945	0.002	-1.48	<0.0001
NOS3	2.7	0.0007	-5.36	0.0034
ASPN	-0.605	0.196	3.93	<0.0001
CTSK	-0.542	0.001	5.12	<0.0001
RUNX2	-0.84	0.005	0.924	<0.0001
LOX	1.16	0.0125	-1.76	<0.0001
LOXL1	0.844	0.0124	0.417	<0.0001
LOXL2	1.79	<0.0001	-1.37	<0.0001
LOXL4	1.18	0.0212	1.88	<0.0001

### 2.3 The osteocyte mechanosome and sex specific differences in both human long bone derived osteoblasts and the mouse osteocyte transcriptome signature

Comparisons of the osteocyte mechanosome (24), with differentially expressed in males and females in the osteocyte transcriptome (26) (Analysis 1) and genes differentially regulated in males and females in human long bone (27) (Analysis 2) revealed several genes in common across datasets. Of the five genes regulated in the mouse transcriptome at 16-weeks (Section 2.1, **Figure 2**), three (TENM4, LOXL1, and SEMA7A) were also regulated by sex in the human long bone derived osteoblasts (Section 2.1, **Figure 4**) (**Supplementary Table 6**). Furthermore, 58 genes were regulated in the mouse osteocyte transcriptome at 26-weeks (Section 2.1) and by sex in the human long bone dataset (**Supplementary Table 7**). Of note, the collagen cross-linking enzyme lysyl oxidase (LOX) and its paralogs, LOX-like-1, 2, and -4 were regulated in the osteocyte mechanosome and either Analysis 1 or 2 (**Figures 4**–**6**). TENM4 was the only regulated gene in the osteocyte mechanosome that was also sex regulated across all datasets (**Tables 2**, **3**).

**Table 3:** Genes regulated in the osteocyte mechanosome and differentially expressed by sex in mouse osteocyte transcriptome selected for discussion within text as mediators linked to pain generation in OA pathology.

Gene	Mechanosome		16- week Mouse osteocyte		26- week Mouse osteocyte	
	log2FC	P(adj)	log2FC	P(adj)	log2FC	P(adj)
TENM4	5.79	0.001	5.7	>0.0001	1.21	0.014
SEMA7A	0.945	0.002	0.945	0.002		
LOXL1	0.844	0.0124	0.844	0.012		
POGK	0.64	0.01	0.64	0.01		
ACP5	-1.23	>0.0001	1.23	>0.0001		
LOX	1.16	0.0125			0.859	0.0257
CTSK	-0.542	0.001			1.18	>0.0001

### 2.4 Analysis 3 - the osteocyte mechanosome and sex specific OA risk factors

Analysis 3 (**Figure 1**), compared genes regulated in the osteocyte mechanosome and by sex in human long bone (Analysis 2) with OA risk loci associated with sex-specific OA and OA pain identified by a GWAS meta-analysis across 826,690 individuals, including 177,517 with OA (28). This study identified 3 sex-specific OA risk loci using a sex-differentiated test of association and a test of heterogeneity in allelic effects, and 11 genes associated with total joint replacement (TJR) surgery which the authors proposed were candidate genes associated with heightened OA pain [**Supplementary Table 5**, (28)].

Of the 3 female specific OA risk loci shown by Boer et al. (28), FANCL, C8orf34, UBAP2 (28), only one gene (FANCL) showed significant down regulation by mechanical loading (P(adj)=0.00087, -0.519-log2FC) in the osteocyte mechanosome. FANCL also showed a significant regulation by sex in the human male and female long bones dataset [SAGD_00129, (27)]. Female long bones showed significantly lower FANCL expression compared with males (P(adj)=0.005, -0.701 - log2FC). Of the 11 genes associated with TJR surgery reflecting heightened OA pain, both PTCH1 (P(adj)=0.02, 1.927 - log2FC), and SERPINA1 (P(adj) 0.007, -3.66 - log2FC) were DEGs in the osteocyte mechanosome, but none showed sex regulation in either the human long bone or mouse osteocyte transcriptome datasets.

Analysis of the 77 OA effector genes published in this GWAS dataset [**Supplementary Table 10**, (28)] revealed 32 effector genes that are mechanically regulated in the osteocyte mechanosome and sex regulated in human long bones (**Supplementary Table 8**). These genes included: CTSK, RUNX2, NOS3 and members of the TGF-β pathway TGFB1, LTBP1 and LTBP3. No GWAS derived effector genes were significantly regulated by sex in the mouse osteocyte transcriptome.

### 2.5 Druggable targets

The genes in the osteocyte mechanosome shown to be sex specific in either GWAS, human long bones or the mouse osteocyte transcriptome were searched on the Drug-Gene Interaction Database to identify potential druggable targets [https://www.dgidb.org, (30)].

In Analysis 1, none of the 5 genes identified in 16-week-old mice were druggable. In 26-week-old mice, 14 of the 58 genes in common represented druggable targets with 9 of these genes having at least one approved drug (**Supplementary Table 9**).

793 of the 3861 genes regulated by mechanical loading in the osteocyte mechanosome and by sex in the human long bone dataset (Analysis 2) represent druggable targets with 580 of these having at least one approved drug (**Supplementary Table 10**). In total this represents 4332 approved drugs due to gene target redundancy. GO term analysis of these druggable targets revealed enrichment for genes associated with the extracellular space and protein phosphorylation and kinase activity. Druggable targets included 107 of the 275 ECM associated genes (442 drugs), 39 of the 93 genes associated with ubiquitin ligase (218 drugs), 18 of the 39 genes associated with immune responses (76 drugs), 54 of the 155 synapse genes (321 drugs), and 2 of the 44 axon guidance genes (1 drug).

In Analysis 3, FANCL, the female specific risk variant for OA (28) that was mechanically downregulated in osteocytes and differentially expressed in male and female human long bones, is also a druggable target with the approved drug Olaparib. Of the 26 GWAS effector genes shown to be significantly regulated by mechanical load in the osteocyte mechanosome and by sex in the human long bone dataset 10 genes had associated drugs with 7 of these being approved.

## 3 Discussion

Combining our transcriptome data of the *in vitro* 3D osteocyte response to pathophysiological mechanical load (24), with published datasets, of osteocyte specific sex-based transcriptome differences (Analysis 1) (26), human long bone explant-derived osteoblast sex-based transcriptome differences (Analysis 2) [repository number 00129 from (33)], and patient sex-specific OA risk factors (Analysis 3) (28), revealed a wide array of sex-regulated genes that are also significantly regulated by pathophysiological loading in osteocytes. Oestrogen deficiency in menopause is thought to contribute to the higher burden of pain experienced by female patients (38). This may involve both the chondroprotective signalling effect of oestrogen (39) as well as its well-established role in protecting bone mass.(14) Despite the high association of oestrogen deficiency predisposing to musculoskeletal pain, a causal link is lacking (8). The disparity between cartilage degradation and pain, and new revelations displaying nociceptor plasticity and invasion of subchondral bone [reviewed in (16)], and the association of BMLs with pain (12), suggests a role for bone in explaining sex differences in OA pain sensation.

LOXL1, SEMA7A and TENM4 were the only differentially expressed genes in the osteocyte mechanosome that were also sex regulated genes common across the 16-week-old mouse transcriptome and the human long bone dataset. TENM4 was the only regulated gene present across all datasets. TENM4 was upregulated by mechanical loading in the osteocyte mechanosome and increased in females in all analyses. TENM4 encodes for Teneurin transmembrane protein 4, a protein important in establishing proper neuronal connectivity during development (40) which has been linked to changes in pain sensitivity (41). Tenm4 mutant mice (Tenm4^em1(IMPC)Tcp^ allele) exhibit sex-specific increased bone mineral content in older female mice [(42); www.mousephenotype.org]. LOX was upregulated by mechanical load in the osteocyte mechanosome, and in the female mouse osteocyte transcriptome at 26 weeks but decreased in female long bones. LOX was not detected in the mouse osteocyte transcriptome at 16 weeks. In addition, LOXL1 was upregulated in the mechanosome, female mouse osteocytes and female long bones. LOXL1 was not detected in mouse osteocyte transcriptome at 26 weeks. LOX and LOXL1 were also highlighted in the STRING protein interaction network analysis of the osteocyte mechanosome combined with sex differences in both human long bones and the osteocyte transcriptome. These enzymes are critical for elastin biogenesis and collagen cross-link formation and play roles in matrix remodelling in normal and disease states (43). Knockouts of LOXL1 have also been shown to induce deterioration of trabecular bone structure in long bones and vertebrae in female mice but not in males (44). Proteolytic activation of LOX is enhanced by the interaction of periostin and BMP1 (45). The sex-specific mechano-regulation of the LOX pathway we have reported links to the findings of Zhou et al. who found POSTN, the gene encoding periostin, to be mechanoresponsive and co-regulated in OA and the osteocyte signature (46). SEMA7A, encoding the neuroimmune axon guidance factor Semaphorin7A was upregulated by load in the osteocyte mechanosome and regulated by sex in our analysis. SEMA7A was down regulated in human female long bones but up regulated in 16-week-old female mouse osteocytes. Semaphorin7A is a signalling ligand that promotes neuron axon elongation and invasion in the developing embryo (47) and is essential in establishing innervation of the dentin-pulp complex (48). CTSK, encoding the lysosomal cysteine protease Cathepsin K a marker of osteoclast bone resorption, was down regulated by mechanical loading in the osteocyte mechanosome but up regulated in both the female human long bone dataset and the female mouse osteocyte transcriptome at 26 weeks. CTSK has been implicated in the pathogenesis of osteoporosis and OA [reviewed in (49)] with inhibition of Cathepsin K delaying OA progression in animal models (50). CTSK was also found to be an OA effector gene (28).

Pathophysiological mechanical loading of osteocytes down regulated the expression of FANCL a ubiquitin ligase previously shown to be associated with female specific risk of hip OA (28). The down regulation of FANCL is associated with cytogenetic instability, hypersensitivity to DNA crosslinking agents, increased chromosomal breakage, and defective DNA repair (51). Both human genetic studies and mouse gene knockouts (52) indicate that loss of function mutations in FANCL, cause premature ovary insufficiency, a condition that leads to early menopause (53). This is of interest as menopausal and post-menopausal females are two times more likely to suffer from joint pain than pre-menopausal females (54, 55). Our data implicates osteocyte response to mechanical loading as a potential mechanism underlying the heightened susceptibility of females with FANCL mutations to OA.

Interestingly, mechanical loading of osteocytes (24) regulated numerous genes associated with bone responses which show differential expression by sex. Bone disease and musculoskeletal disease were enriched in disease gene associated analysis of the protein interactions of the osteocyte mechanosome when combined with the sex regulated genes in human long bones. GO:term enrichment showed that both in the mouse osteocyte data and in human long bone data the mechanosome revealed regulated genes associated with bone mineralisation and bone resorption that were differentially expressed by sex. The osteocyte mechanosome and human long bone sex differences dataset also showed regulation of RUNX2 an essential transcription factor in osteoblast differentiation and an OA effector gene in GWAS analysis (28). Mechanical load down regulated RUNX2, whereas RUNX2 was upregulated in females. This data suggests that the regulation of bone formation and resorption by osteocytes in response to mechanical loading is different in males and females.

39 genes associated with immune responses showed co-regulation by mechanical load in the osteocyte mechanosome and by sex in the human long bone dataset. Differential expression of genes in females also showed enrichment for the rheumatoid arthritis pathway. These data show that pathophysiological loading of osteocytes causes immune factor expression that is significantly differentially expressed in females. Interestingly, NOS3 was identified by Boer et al. as an OA effector gene (28) and is upregulated by mechanical load and in male human long bones. This endothelium isoform of nitric oxide synthase is the predominant constitutive isoform of NOS within bone (56), mechanically regulated in osteocytes (57) and expressed in human osteocytes *in vivo* (58). It is an important mediator of inflammatory signalling (59), and plays a role in mediating oestrogen-induced bone formation in female mice (58). These data suggest that differences in inflammatory and immune signalling in the mechanical responses of females may drive differential immune signalling leading to higher nociceptive signalling in females.

The TGF-β signalling pathway was also shown to be differentially expressed in STRING pathway enrichment analysis of the osteocyte mechanosome and human long bone sex differences combined datasets. Members of this pathway were identified by Boer et al. as effector genes in a large GWAS analysis (28). TGF-β is a pleiotropic cytokine that is only active in the healthy joint after mechanical loading. In the OA joint, TGF-β signalling is greatly enhanced (60). Sexual dimorphism in TGF-β responses was demonstrated in mice, where osteocyte specific knockout of the TGF-β receptor II increased subchondral bone thickening in male but not female mice and was associated with cartilage degeneration (61). The sex-regulation of the TGF-β pathway shown in this analysis reinforces the evidence that differential inflammatory and immune signalling in females may drive differences in OA progression and pain. Asporin acts as a negative regulator of chondrogenesis by inhibiting TGF-β function (62). Recently, ASPN has been shown to be a disease-relevant gene, contributing to subchondral bone remodelling in OA (46). ASPORIN (ASPN) is a small leucine-rich repeat proteoglycan (SLRP) with polymorphisms that are strongly associated with OA (63). It directly binds TGF-β1 and subsequently collagen, playing a role in collagen fibrillogenesis and metabolism (64, 65). ASPN more highly expressed in female human long bones compared to equivalent male samples but was not significantly regulated in the mouse osteocyte transcriptome by sex.

Comparison of the mechanosome with genes differentially expressed in human male and female long bones highlighted pathways involved in neuronal activity, ECM, immune response, and identified associations with many painful musculoskeletal diseases involving bone and neuropathies. 93 genes associated with ubiquitin protein ligase binding were significantly regulated by sex in the human long bone dataset and significantly regulated in our osteocyte mechanical loading dataset. No genes associated with ubiquitin function were differentially expressed between males and females in the sex specific osteocyte transcriptome when combined the osteocyte mechanosome. Ubiquitin disfunction in OA is an emerging pathway in driving pathology especially in regulating the apoptosis and hypertrophic differentiation of chondrocytes (66). It is also likely that changes in ubiquitin function contribute to bone changes in OA as it plays an important role in regulating bone remodelling as well as osteocyte apoptosis (67) with proteosome inhibitors effectively reducing bone turnover and increasing osteocyte viability in multiple myeloma (68).

155 genes associated with the synapse, 4 genes associated with the sensory perception of pain and 44 genes associated with axon guidance and cell projection were significantly regulated by osteocyte mechanical loading and by sex in the human long bone dataset. The regulation of this number of neuronally associated genes in both datasets provides evidence that the nociceptor bone interface, and the response of osteocytes to pathological load differs in female OA patients compared to that of males. TENM4 and SEMA7A in both the sex differential human long bone and mouse derived osteocyte signature dataset shows that in both mouse models and in human patients, differential axon guidance signalling in males and females may result in differing levels of nociceptor plasticity and sensitivity in females. All the semaphorin signalling ligands displayed higher differential expression in males in human long bones. Axon guidance signalling factors have been shown to regulate sensory nerve sprouting and invasion in mouse models (69) and to regulate the membrane potential of sensory neurons (70), with signalling cascades that integrate to the signalling of NGF (71). The axon guidance signalling pathway has also recently been reported by Zhou et al. to be a significantly enriched pathway in the 223 main contributory genes between the medial OA subchondral bone and lateral plateau in mice OA models (46). In contrast we saw no differences in NGF signalling in our analyses.

Significantly more DEGs were detected in ageing 26-week-old male and female mouse osteocytes and the osteocyte mechanosome compared with 16-week-old mice. Hyperalgesia lasts longer and is more pronounced in older rats, with aged females exhibiting the most impaired responses (72). Age also impacts OA pain in humans with clinical studies revealing older woman to have more chronic pain (73, 74). (75) hypothesised that brain changes observed in the early stages of monosodium iodoacetate-induced OA in rats may account for the increased risk for ageing females to develop chronic pain. This is supported by our String analysis which revealed the significant enrichment of neurodegenerative diseases in females. GO term analysis revealed regulation of DEGS involved in the immune response in both the mechanosome and osteocytes of aging mice. Studies have linked higher pain scores and lower pain thresholds in woman to enhanced inflammatory responses (76–78). In addition, sex differences exist in the relationship between individual systemic markers of inflammation and pain in knee osteoarthritis (79, 80).

## 4 Limitations and conclusions

The greatly reduced number of sex-regulated genes in the mouse osteocyte transcriptome data raises questions as to the similarities between bone-nerve interactions in mouse models compared to patients. It is established that female and male bone display many differences in physiology and intricate associations with the nervous system. Recent research has shown that there are large differences between the peripheral sensory nervous systems in mice and humans (81). Sensory nerve gene expression, molecular fingerprint, and sensory nerve sub populations have been shown to be different between mouse models and human patients. This analysis therefore raises the possibility that the differential expression of factors that influence sensory nerve changes in animal models limit their effectiveness in studying nociceptor changes in OA. Since the human sex specific long bone data was based on bone explant derived osteoblasts rather than osteocytes *in vivo*, it is also possible that sex specific differences from the human data set are not osteocyte specific.

This analysis has shown a wide array of factors regulated in osteocytes by mechanical loading that are differentially expressed by sex and influence innervation, neural activity and bone remodelling associated with OA pain. It remains to be determined whether these sex specific differences in responses would differentially effect nociceptor populations in males and females. To test this, sex-specific differences in receptor complexes or susceptibility to the differences in osteocyte derived neural signalling would need to be investigated.

Our comparison of the osteocyte mechanosome to published data reflecting sex specific gene expression and susceptibility to OA pain has highlighted pain related pathways potentially responsible for elevated pain susceptibility in females with osteoarthritis. The large number of approved drugs available to target these pathways reveals a great opportunity to modulate mechanically driven osteoarthritic pain particularly in susceptible females.

## Data Availability

The original contributions presented in the study are included in the article/**Supplementary Material**. Further inquiries can be directed to the corresponding author.

## Supplementary material

Supplementary Figure 1 Bar plot of the significantly enriched GO: Terms produced by GO:term enrichment of genes differentially expressed in both the osteocyte mechanosome and by sex in osteocytes from 16-week-old skeletally mature mice. Bar colour is representative of adjusted p value. Bar length represents the number of genes associated with each GO:term within the dataset.

Supplementary Figure 2 Bar plot of the significantly enriched GO: Terms produced by GO:term enrichment of genes differentially expressed in both the osteocyte mechanosome and by sex in osteocytes from 26-week-old skeletally mature mice. Bar colour is representative of adjusted p value. Bar length represents the number of genes associated with each GO:term within the dataset.

Supplementary Figure 3 Protein-protein interactions with high confidence interaction score within significantly regulated osteocyte mechanosome and sex differences within mouse osteocyte transcriptome dataset. Coloured nodes represent genes within the dataset within the first shell of interactions. Node content represents predicted protein 3D structure. Connecting lines represent the established protein-protein associations. Line thickness represents the confidence score of these interactions.

Supplementary Figure 4 Bar plot of the significantly enriched GO: Terms produced by GO:term enrichment of genes regulated by sex in human long bone derived osteoblasts and mechanical loading in osteocytes. **(A)** All enriched GO: Terms, **(B)** Enriched biological process GO: Terms, **(C)** Enriched Cellular compartment GO: Terms, **(D)** Enriched molecular function GO: Terms. Bar colour is representative of adjusted p value. Bar length represents the number of genes associated with each GO:term within the dataset.

Supplementary Figure 5 Protein-protein interactions with high confidence interaction score within significantly regulated osteocyte mechanosome and sex differences within human long bone derived osteoblast dataset. Coloured nodes represent genes within the dataset within the first shell of interactions. Node content represents predicted protein 3D structure. Connecting lines represent the established protein-protein associations. Line thickness represents the confidence score of these interactions.
