# Supplementary figures and images for: Osteocytes contribute to sex-specific differences in osteoarthritic pain
Source: Front Endocrinol (Lausanne). 2024 Nov 7;15:1480274. doi: 10.3389/fendo.2024.1480274 (PMC11579924; doi:10.3389/fendo.2024.1480274)

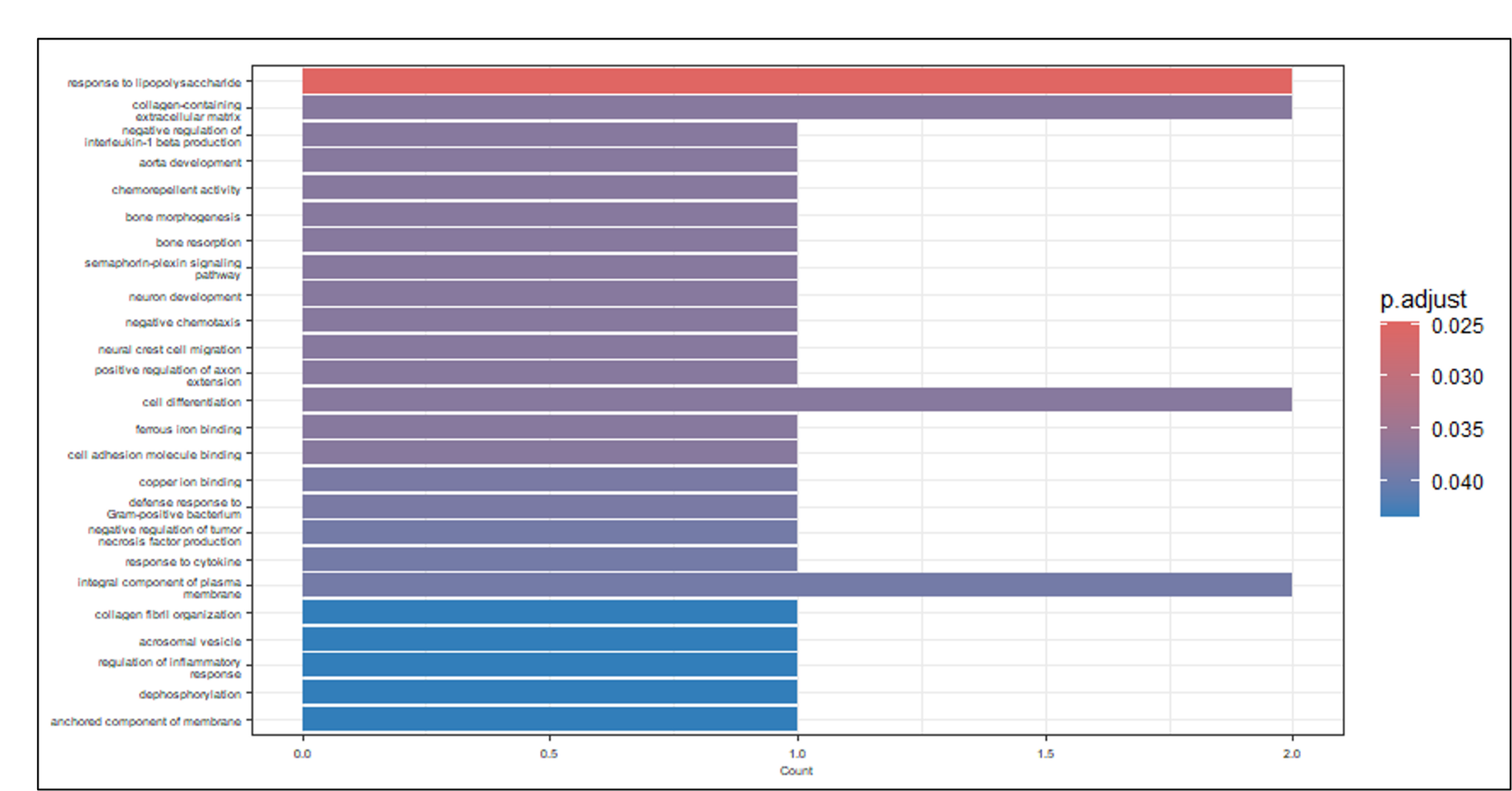

Supplement: Supplementary Figure 1 — Bar plot of the significantly enriched GO: Terms produced by GO:term enrichment of genes differentially expressed in both the osteocyte mechanosome and by sex in osteocytes from 16-week-old skeletally mature mice. Bar colour is representative of adjusted p value. Bar length represents the number of genes associated with each GO:term within the dataset. [file Image1.png]

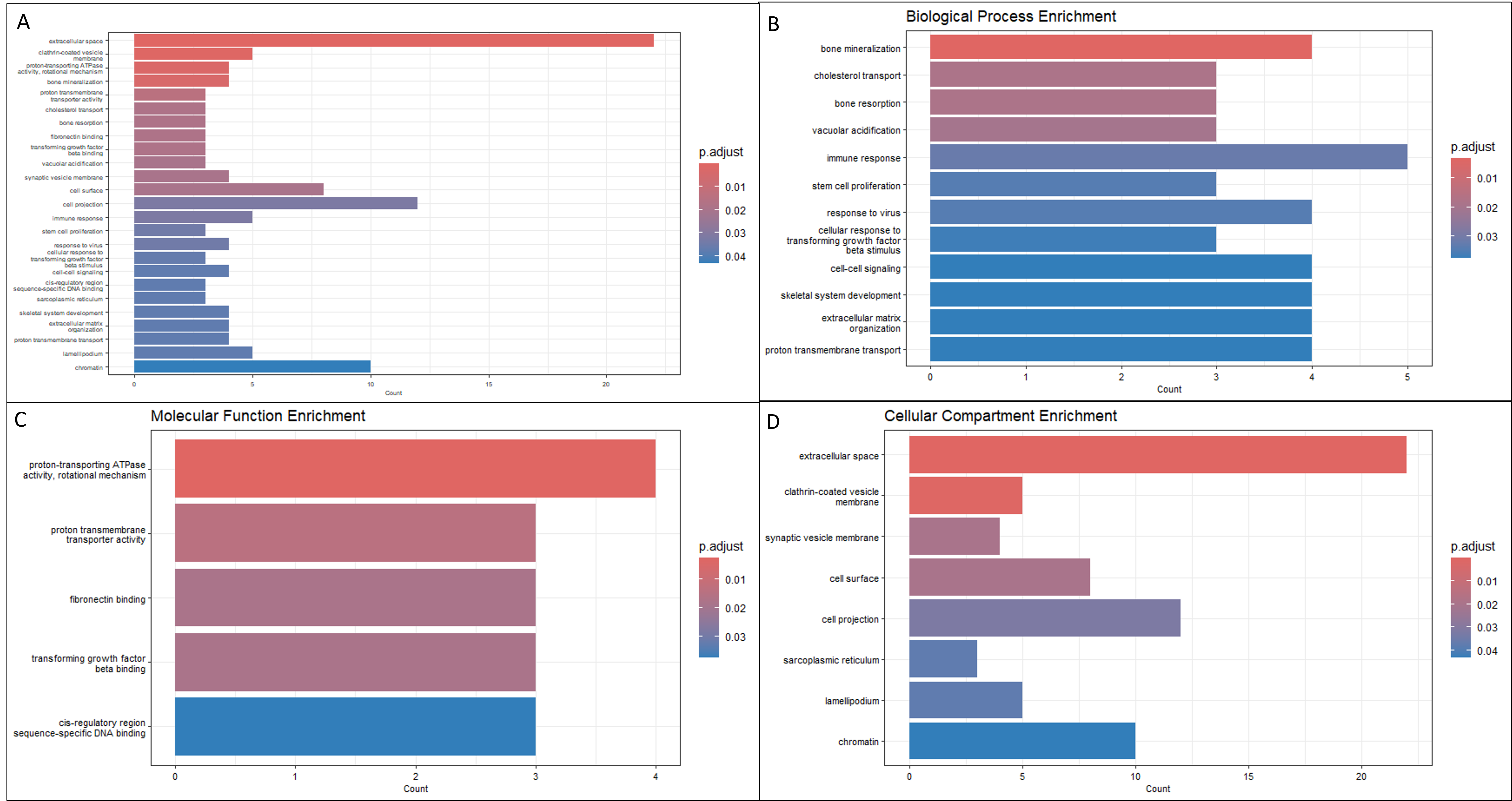

Supplement: Supplementary Figure 2 — Bar plot of the significantly enriched GO: Terms produced by GO:term enrichment of genes differentially expressed in both the osteocyte mechanosome and by sex in osteocytes from 26-week-old skeletally mature mice. Bar colour is representative of adjusted p value. Bar length represents the number of genes associated with each GO:term within the dataset. [file Image2.png]

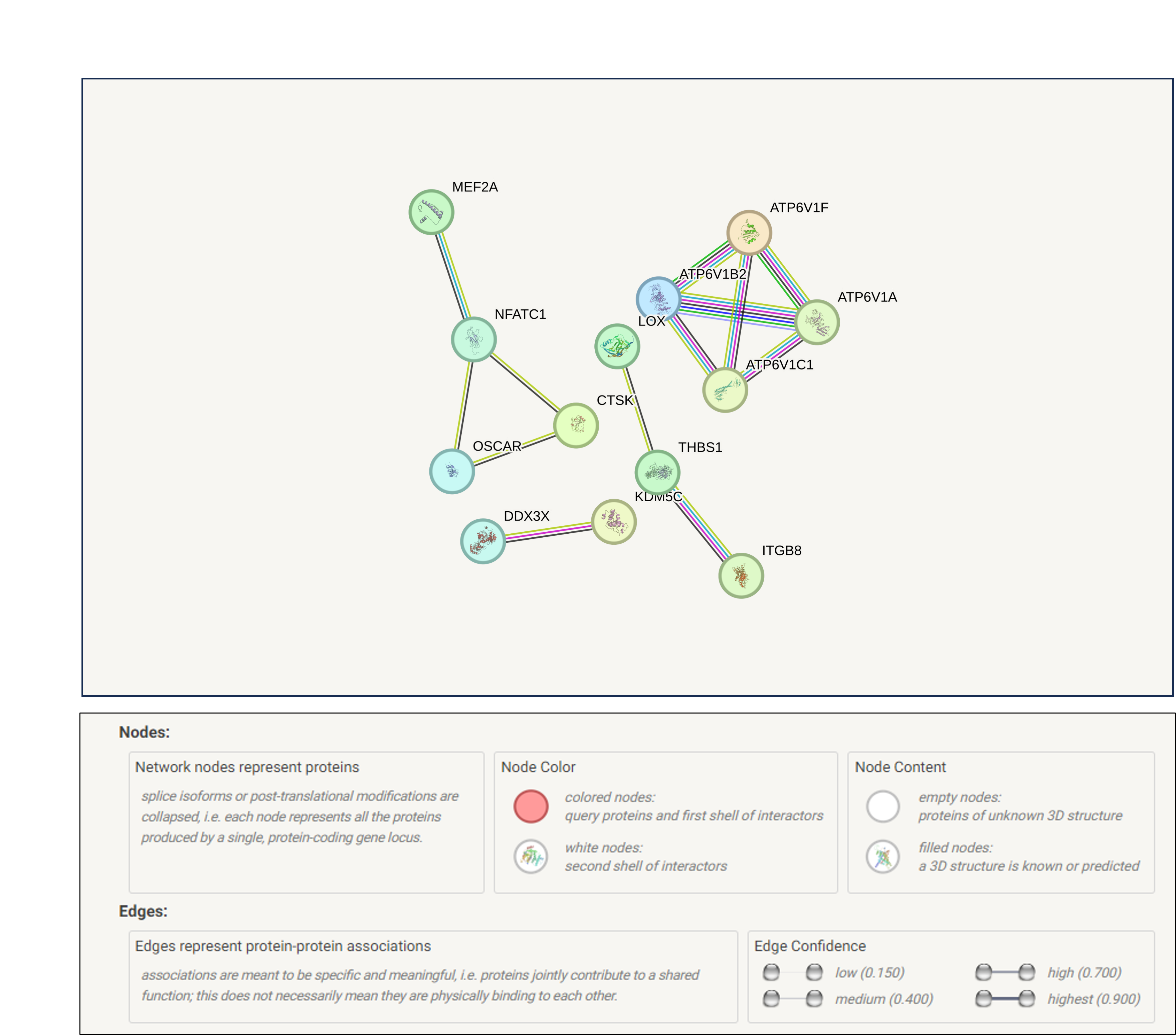

Supplement: Supplementary Figure 3 — Protein-protein interactions with high confidence interaction score within significantly regulated osteocyte mechanosome and sex differences within mouse osteocyte transcriptome dataset. Coloured nodes represent genes within the dataset within the first shell of interactions. Node content represents predicted protein 3D structure. Connecting lines represent the established protein-protein associations. Line thickness represents the confidence score of these interactions. [file Image3.png]

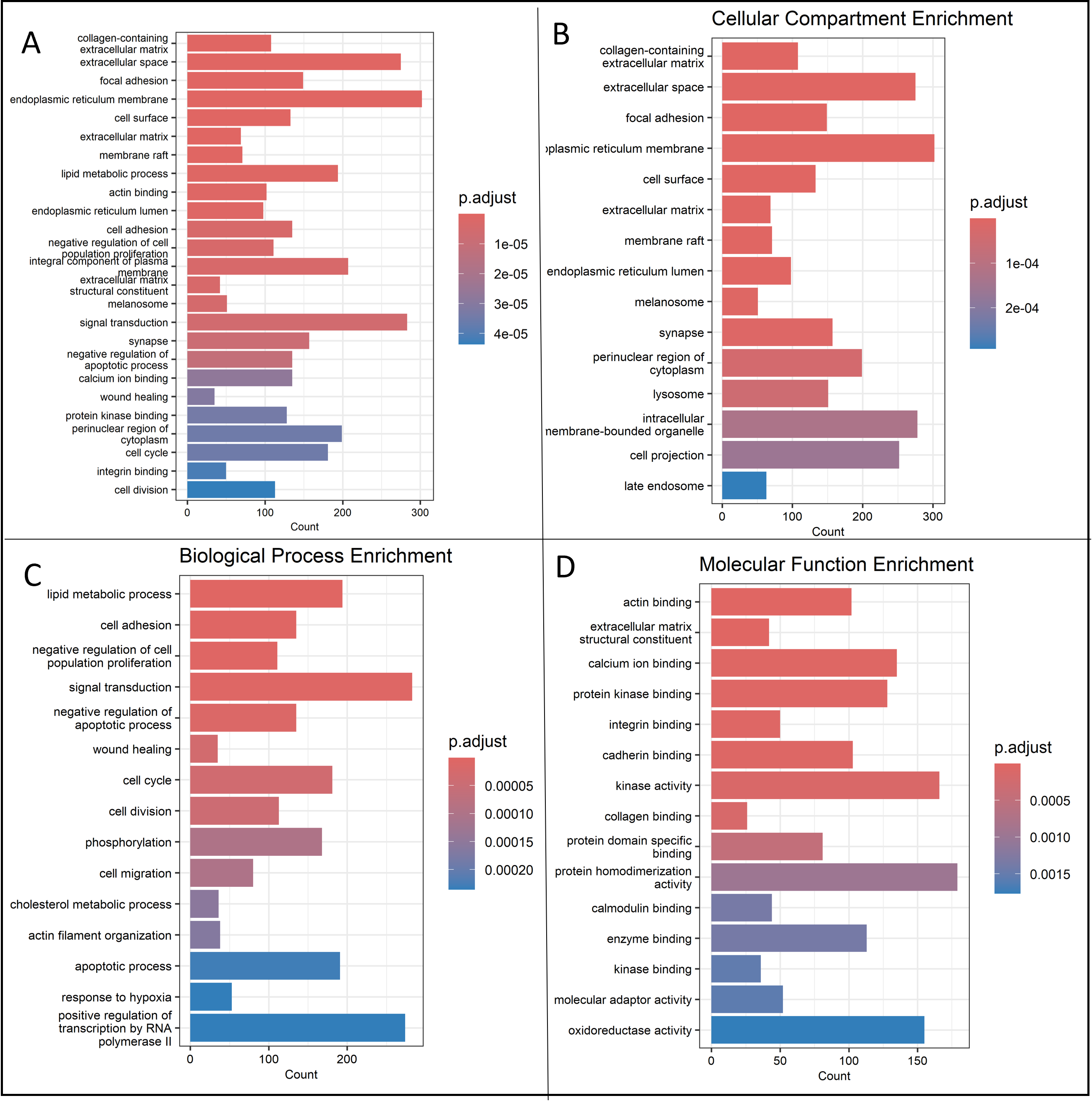

Supplement: Supplementary Figure 4 — Bar plot of the significantly enriched GO: Terms produced by GO:term enrichment of genes regulated by sex in human long bone derived osteoblasts and mechanical loading in osteocytes. (A) All enriched GO: Terms, (B) Enriched biological process GO: Terms, (C) Enriched Cellular compartment GO: Terms, (D) Enriched molecular function GO: Terms. Bar colour is representative of adjusted p value. Bar length represents the number of genes associated with each GO:term within the dataset. [file Image4.png]

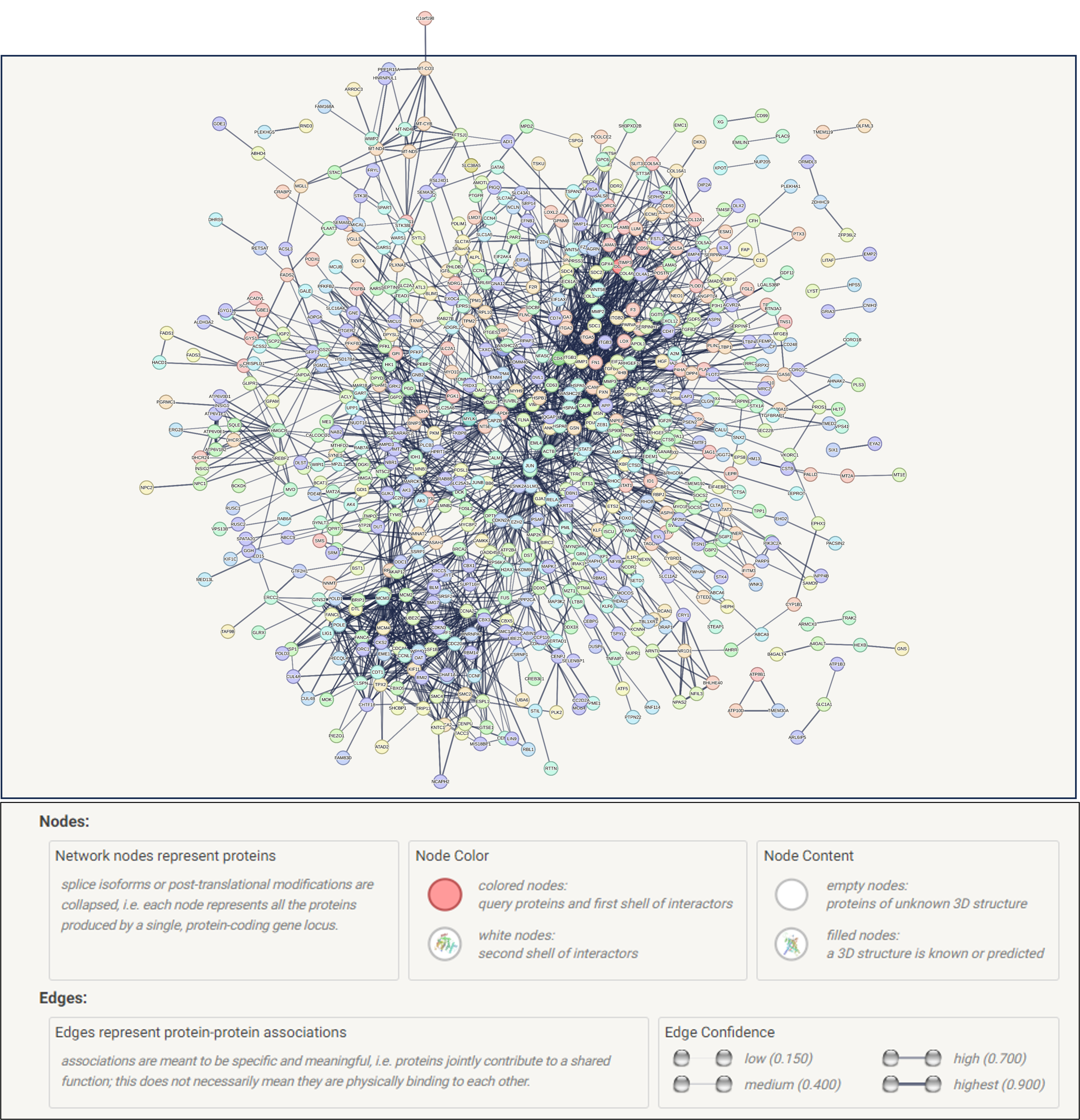

Supplement: Supplementary Figure 5 — Protein-protein interactions with high confidence interaction score within significantly regulated osteocyte mechanosome and sex differences within human long bone derived osteoblast dataset. Coloured nodes represent genes within the dataset within the first shell of interactions. Node content represents predicted protein 3D structure. Connecting lines represent the established protein-protein associations. Line thickness represents the confidence score of these interactions. [file Image5.png]
