# Supplementary material for: Osteocytes contribute to sex-specific differences in osteoarthritic pain
Source: Front Endocrinol (Lausanne). 2024 Nov 7;15:1480274. doi: 10.3389/fendo.2024.1480274 (PMC11579924; doi:10.3389/fendo.2024.1480274)
Supplement: Supplementary file 14 [file Table9.docx]

Supplementary Table 9: Approved drugs that potentially target mechanically regulated genes (Gilbert et al 2024) that are differentially expressed in osteocytes from 26 week old male and female mice (Youlten et al., 2021) from dgidb.org accessed 20.12.23 and the female specific specific risk variant for OA (Boer et al., 2021) that was mechanically regulated and differentially expressed in male and female long bones.

| **gene** | **drug** | **indication** |
| --- | --- | --- |
| **HEXB** | TAMOXIFEN | Hormonal,Antineoplastic Agents |
| **HEXB** | CISPLATIN |  |
| **HEXB** | CORTISONE |  |
| **HEXB** | GENTAMICIN |  |
| **HEXB** | RABEPRAZOLE | Proton pump inhibitor |
| **HEXB** | OMEPRAZOLE | Proton pump inhibitor,antiulcer agent |
| **HEXB** | ESTRAMUSTINE |  |
| **CAMK2G** | HALOPERIDOL DECANOATE | Antipsychotic Agents |
| **CAMK2G** | BOSUTINIB | antineoplastic agent |
| **CAMK2G** | LITHIUM |  |
| **CAMK2G** | GEFITINIB | antineoplastic agent |
| **TUBB6** | VINBLASTINE | Antineoplastic Agents |
| **TUBB6** | MIRVETUXIMAB SORAVTANSINE |  |
| **TUBB6** | VINORELBINE | antineoplastic agent |
| **TUBB6** | VINFLUNINE |  |
| **TUBB6** | DOCETAXEL ANHYDROUS | antineoplastic agent |
| **TUBB6** | ADO-TRASTUZUMAB EMTANSINE | antineoplastic agent |
| **TUBB6** | VINBLASTINE SULFATE |  |
| **TUBB6** | VINORELBINE TARTRATE |  |
| **TUBB6** | VINCRISTINE | antineoplastic agent |
| **TUBB6** | POLATUZUMAB VEDOTIN |  |
| **TUBB6** | PACLITAXEL | for treatment of peripheral arterial disease (PAD),DMARD,antiinflammatory agent,antineoplastic agent |
| **TUBB6** | TISOTUMAB VEDOTIN-TFTV |  |
| **TUBB6** | CURCUMIN |  |
| **TUBB6** | BRENTUXIMAB VEDOTIN | antineoplastic agent |
| **TUBB6** | ERIBULIN MESYLATE |  |
| **TUBB6** | IXABEPILONE | antineoplastic agent |
| **TUBB6** | CABAZITAXEL | antineoplastic agent |
| **TUBB6** | VINCRISTINE SULFATE |  |
| **TUBB6** | VORINOSTAT | antineoplastic agent |
| **TUBB6** | BELANTAMAB MAFODOTIN-BLMF |  |
| **TUBB6** | ENFORTUMAB VEDOTIN-EJFV |  |
| **TUBB6** | EPOTHILONE D | antineoplastic agent |
| **TUBB6** | PODOFILOX | Phytogenic; Keratolytic Agents,Antineoplastic Agents |
| **TUBB6** | COLCHICINE | for treatment of gout |
| **FZD1** | NICLOSAMIDE |  |
| **PRKAG2** | HYDROCHLOROTHIAZIDE | antihypertensive agent |
| **PRKAG2** | METFORMIN | antidiabetic |
| **A2M** | ALPROSTADIL | for treatment of sexual dysfunction in women,for treatment of erectile dysfunction |
| **A2M** | THROMBIN | topical tissue sealant |
| **A2M** | TECHNETIUM TC 99M SUCCIMER |  |
| **NFATC1** | PREDNISONE | corticosteroid,antiinflammatory agent |
| **NFATC1** | CYCLOSPORINE | opthalmological agent,immunosuppressant |
| **NFATC1** | MYCOPHENOLATE | immunosuppressant |
| **KDM5C** | EVEROLIMUS | immunosuppressant |
| **KDM5C** | SUNITINIB | antineoplastic agent |
| **THBS1** | SIMVASTATIN | antidyslipidaemic agent,anticholesterolaemic agent,antihypertensive agent |
| **THBS1** | PIOGLITAZONE HYDROCHLORIDE | antidiabetic |
| **THBS1** | VITAMIN A |  |
| **THBS1** | LOVASTATIN | anticholesterolaemic agent |
| **THBS1** | DECITABINE | antineoplastic agent |
| **THBS1** | CAMPTOTHECIN | antineoplastic agent |
| **THBS1** | TRETINOIN | for treatment of acne |
| **THBS1** | METHYLCELLULOSE |  |
| **FANCL** | OLAPARIB |  |

**Supplementary Table ?:** Druggable targets (approved and not approved) from mechanically regulated genes (Gilbert et al) identified as differentially expressed in osteocytes from 26 month old male and female mice (Youlten) from dgidb.org accessed 20.12.24

| **gene** | **drug** | **regulatory approval** | **indication** |
| --- | --- | --- | --- |
| **IGFBP6** | ANTISERUM | Not Approved |  |
| **HEXB** | TAMOXIFEN | Approved | Hormonal,Antineoplastic Agents |
| **HEXB** | CISPLATIN | Approved |  |
| **HEXB** | CORTISONE | Approved |  |
| **HEXB** | GENTAMICIN | Approved |  |
| **HEXB** | RABEPRAZOLE | Approved | Proton pump inhibitor |
| **HEXB** | OMEPRAZOLE | Approved | Proton pump inhibitor,antiulcer agent |
| **HEXB** | AMINOGLYCOSIDE ANTIBIOTIC | Not Approved |  |
| **HEXB** | THYROXINE | Not Approved |  |
| **HEXB** | ESTRAMUSTINE | Approved |  |
| **HEXB** | CHEMBL:CHEMBL257158 | Not Approved |  |
| **ITGB8** | ABITUZUMAB | Not Approved |  |
| **CAMK2G** | MEDICATION | Not Approved |  |
| **CAMK2G** | AZD-1080 | Not Approved |  |
| **CAMK2G** | ILORASERTIB | Not Approved |  |
| **CAMK2G** | HALOPERIDOL DECANOATE | Approved | Antipsychotic Agents |
| **CAMK2G** | SP-600125 | Not Approved |  |
| **CAMK2G** | LY-2090314 | Not Approved |  |
| **CAMK2G** | CYC-116 | Not Approved |  |
| **CAMK2G** | SNS-314 | Not Approved | antineoplastic agent |
| **CAMK2G** | NVP-TAE684 | Not Approved |  |
| **CAMK2G** | SOTRASTAURIN | Not Approved |  |
| **CAMK2G** | GW441756X | Not Approved |  |
| **CAMK2G** | DOVITINIB | Not Approved | antineoplastic agent |
| **CAMK2G** | CENISERTIB | Not Approved | antineoplastic agent |
| **CAMK2G** | LINIFANIB | Not Approved | antineoplastic agent |
| **CAMK2G** | BOSUTINIB | Approved | antineoplastic agent |
| **CAMK2G** | LITHIUM | Approved |  |
| **CAMK2G** | GEFITINIB | Approved | antineoplastic agent |
| **CAMK2G** | BAY 61-3606 | Not Approved |  |
| **CAMK2G** | ANTIDEPRESSANT AGENT | Not Approved |  |
| **CAMK2G** | PF-562271 | Not Approved |  |
| **CAMK2G** | TAMATINIB | Not Approved |  |
| **TUBB6** | BIVATUZUMAB MERTANSINE | Not Approved |  |
| **TUBB6** | BMS-275183 | Not Approved |  |
| **TUBB6** | PINATUZUMAB VEDOTIN | Not Approved |  |
| **TUBB6** | CHEMBL:CHEMBL1935538 | Not Approved |  |
| **TUBB6** | VINBLASTINE | Approved | Antineoplastic Agents |
| **TUBB6** | CANTUZUMAB MERTANSINE | Not Approved |  |
| **TUBB6** | CHEMBL:CHEMBL2036124 | Not Approved |  |
| **TUBB6** | NOCODAZOLE | Not Approved |  |
| **TUBB6** | PACLITAXEL POLIGLUMEX | Not Approved |  |
| **TUBB6** | MAYTANSINE | Not Approved |  |
| **TUBB6** | AZINTUXIZUMAB VEDOTIN | Not Approved |  |
| **TUBB6** | COMBRETASTATIN A4 | Not Approved |  |
| **TUBB6** | CHEMBL:CHEMBL1795737 | Not Approved |  |
| **TUBB6** | CHEMBL:CHEMBL2036119 | Not Approved |  |
| **TUBB6** | MIRVETUXIMAB SORAVTANSINE | Approved |  |
| **TUBB6** | VERUBULIN | Not Approved |  |
| **TUBB6** | VINORELBINE | Approved | antineoplastic agent |
| **TUBB6** | PLOCABULIN | Not Approved |  |
| **TUBB6** | VINFLUNINE | Approved |  |
| **TUBB6** | FOSBRETABULIN DISODIUM | Not Approved |  |
| **TUBB6** | GLEMBATUMUMAB VEDOTIN | Not Approved |  |
| **TUBB6** | RG-7636 | Not Approved |  |
| **TUBB6** | AGS-16C3F | Not Approved |  |
| **TUBB6** | INDIBULIN | Not Approved |  |
| **TUBB6** | PLINABULIN | Not Approved |  |
| **TUBB6** | MILATAXEL | Not Approved | antineoplastic agent |
| **TUBB6** | DOCETAXEL ANHYDROUS | Approved | antineoplastic agent |
| **TUBB6** | T-900607 | Not Approved |  |
| **TUBB6** | ADO-TRASTUZUMAB EMTANSINE | Approved | antineoplastic agent |
| **TUBB6** | LEXIBULIN | Not Approved |  |
| **TUBB6** | LADIRATUZUMAB VEDOTIN | Not Approved |  |
| **TUBB6** | LORVOTUZUMAB MERTANSINE | Not Approved |  |
| **TUBB6** | SAGOPILONE | Not Approved |  |
| **TUBB6** | PACLITAXEL DOCOSAHEXAENOIC ACID | Not Approved |  |
| **TUBB6** | DAVUNETIDE | Not Approved |  |
| **TUBB6** | VINBLASTINE SULFATE | Approved |  |
| **TUBB6** | SOFITUZUMAB VEDOTIN | Not Approved |  |
| **TUBB6** | VINORELBINE TARTRATE | Approved |  |
| **TUBB6** | APRUTUMAB IXADOTIN | Not Approved |  |
| **TUBB6** | VINCRISTINE | Approved | antineoplastic agent |
| **TUBB6** | ZAMPANOLIDE | Not Approved |  |
| **TUBB6** | ANG 1005.00 | Not Approved | antineoplastic agent |
| **TUBB6** | CHEMBL:CHEMBL453818 | Not Approved |  |
| **TUBB6** | POLATUZUMAB VEDOTIN | Approved |  |
| **TUBB6** | ARX-788 | Not Approved |  |
| **TUBB6** | PACLITAXEL | Approved | for treatment of peripheral arterial disease (PAD),DMARD,antiinflammatory agent,antineoplastic agent |
| **TUBB6** | TISOTUMAB VEDOTIN-TFTV | Approved |  |
| **TUBB6** | TELISOTUZUMAB VEDOTIN | Not Approved |  |
| **TUBB6** | RG-7600 | Not Approved |  |
| **TUBB6** | CURCUMIN | Approved |  |
| **TUBB6** | BRENTUXIMAB VEDOTIN | Approved | antineoplastic agent |
| **TUBB6** | VANDORTUZUMAB VEDOTIN | Not Approved |  |
| **TUBB6** | ERIBULIN MESYLATE | Approved |  |
| **TUBB6** | TUSAMITAMAB RAVTANSINE | Not Approved |  |
| **TUBB6** | IXABEPILONE | Approved | antineoplastic agent |
| **TUBB6** | KOS-1584 | Not Approved |  |
| **TUBB6** | INDUSATUMAB VEDOTIN | Not Approved |  |
| **TUBB6** | ASG-5ME | Not Approved |  |
| **TUBB6** | OMBRABULIN | Not Approved | antineoplastic agent |
| **TUBB6** | CABAZITAXEL | Approved | antineoplastic agent |
| **TUBB6** | PF-06263507 | Not Approved |  |
| **TUBB6** | PATUPILONE | Not Approved |  |
| **TUBB6** | VINCRISTINE SULFATE | Approved |  |
| **TUBB6** | LIFASTUZUMAB VEDOTIN | Not Approved |  |
| **TUBB6** | ENAPOTAMAB VEDOTIN | Not Approved |  |
| **TUBB6** | CROLIBULIN | Not Approved |  |
| **TUBB6** | SOBLIDOTIN | Not Approved |  |
| **TUBB6** | LARGAZOLE | Not Approved |  |
| **TUBB6** | CYCLOSTREPTIN | Not Approved |  |
| **TUBB6** | LAROTAXEL | Not Approved |  |
| **TUBB6** | DOLASTATIN-10 | Not Approved |  |
| **TUBB6** | VORINOSTAT | Approved | antineoplastic agent |
| **TUBB6** | BELANTAMAB MAFODOTIN-BLMF | Approved |  |
| **TUBB6** | ABT-751 | Not Approved |  |
| **TUBB6** | PRALUZATAMAB RAVTANSINE | Not Approved |  |
| **TUBB6** | ENFORTUMAB VEDOTIN-EJFV | Approved |  |
| **TUBB6** | FOSBRETABULIN TROMETHAMINE | Not Approved |  |
| **TUBB6** | EPOTHILONE D | Approved | antineoplastic agent |
| **TUBB6** | MAYTANSINOL | Not Approved |  |
| **TUBB6** | RG-7841 | Not Approved |  |
| **TUBB6** | PODOFILOX | Approved | Phytogenic; Keratolytic Agents,Antineoplastic Agents |
| **TUBB6** | COLCHICINE | Approved | for treatment of gout |
| **FZD1** | NICLOSAMIDE | Approved |  |
| **FZD1** | CHEMBL:CHEMBL464249 | Not Approved |  |
| **FZD1** | VANTICTUMAB | Not Approved |  |
| **FZD1** | CHEMBL:CHEMBL593726 | Not Approved |  |
| **PRKAG2** | ACADESINE | Not Approved |  |
| **PRKAG2** | SAPONARIN | Not Approved |  |
| **PRKAG2** | HYDROCHLOROTHIAZIDE | Approved | antihypertensive agent |
| **PRKAG2** | METFORMIN | Approved | antidiabetic |
| **APOD** | RETINOIC ACID AGENT | Not Approved |  |
| **CTSK** | BALICATIB | Not Approved |  |
| **CTSK** | ODANACATIB | Not Approved | antiosteoporotic agent |
| **CTSK** | RELACATIB | Not Approved | antiosteoporotic agent |
| **CTSK** | CHEMBL:CHEMBL1085282 | Not Approved | antirheumatic agent |
| **CTSK** | MIV-701 | Not Approved | for treatment of osteoporosis |
| **CTSK** | ANTISENSE OLIGONUCLEOTIDES | Not Approved |  |
| **CTSK** | CHEMBL:CHEMBL2316602 | Not Approved |  |
| **CTSK** | HEPARAN SULFATE | Not Approved |  |
| **CTSK** | ONO-5334 | Not Approved |  |
| **A2M** | COBALT | Not Approved |  |
| **A2M** | ALPROSTADIL | Approved | for treatment of sexual dysfunction in women,for treatment of erectile dysfunction |
| **A2M** | THROMBIN | Approved | topical tissue sealant |
| **A2M** | TECHNETIUM TC 99M SUCCIMER | Approved |  |
| **NFATC1** | PREDNISONE | Approved | corticosteroid,antiinflammatory agent |
| **NFATC1** | CYCLOSPORINE | Approved | opthalmological agent,immunosuppressant |
| **NFATC1** | MYCOPHENOLATE | Approved | immunosuppressant |
| **KDM5C** | EVEROLIMUS | Approved | immunosuppressant |
| **KDM5C** | SUNITINIB | Approved | antineoplastic agent |
| **MYLK** | TOZASERTIB | Not Approved | antineoplastic agent |
| **THBS1** | PROTEIN KINASE C INHIBITOR | Not Approved |  |
| **THBS1** | TSP-1 MIMETIC FUSION PROTEIN CVX-045 | Not Approved |  |
| **THBS1** | SIMVASTATIN | Approved | antidyslipidaemic agent,anticholesterolaemic agent,antihypertensive agent |
| **THBS1** | PIOGLITAZONE HYDROCHLORIDE | Approved | antidiabetic |
| **THBS1** | ABT-510 | Not Approved | antineoplastic agent |
| **THBS1** | VITAMIN A | Approved |  |
| **THBS1** | LOVASTATIN | Approved | anticholesterolaemic agent |
| **THBS1** | DECITABINE | Approved | antineoplastic agent |
| **THBS1** | ACTH | Not Approved |  |
| **THBS1** | CAMPTOTHECIN | Approved | antineoplastic agent |
| **THBS1** | TRETINOIN | Approved | for treatment of acne |
| **THBS1** | GP-120 ANTIGEN | Not Approved |  |
| **THBS1** | VESNARINONE | Not Approved |  |
| **THBS1** | METHYLCELLULOSE | Approved |  |
